# CXCL2 acts as a prognostic biomarker and associated with immune infiltrates in stomach adenocarcinoma

**DOI:** 10.1097/MD.0000000000031096

**Published:** 2022-10-21

**Authors:** Jingxin Zhang, Wenji Hou, Junbo Zuo, Zhenhua Huang, Xin Ding, Xuefeng Bu

**Affiliations:** a Department of General Surgery, Affiliated People’s Hospital of Jiangsu University, Zhenjiang, China.

**Keywords:** bioinformatics analysis, biomarker, CXCL2, immune infiltrate, STAD

## Abstract

STAD ranked 5th most common in the incidence of malignant tumors and 3rd most common in the death rate of cancer worldwide. CXC chemokines affect the biological progress of various tumors, resulting in therapeutic failure. The role of CXCL2 in STAD was still a mystery.

**Methods:**

The expression, prognostic value, and clinical function of CXCL2 were analyzed using several online bioinformatics tools and clinical tissues.

**Results:**

CXCL2 level was significantly upregulated in STAD tissues. Strong correlation was obtained between CXCL2 level and immune cells as well as immune biomarkers. High CXCL2 expression in STAD was correlated with a favorable prognosis. Further analysis revealed that CXCL2, pTNM stage and age were independent factors affecting the prognosis of STAD patients. A predictive nomogram indicated that the calibration plots for the 1-year, 3-year and 5-year OS rates were predicted relatively well compared with an ideal model in the entire cohort. Validation analysis revealed that CXCL2 expression was upregulated in STAD and high CXCL2 level had a better overall survival. CXCL2 was associated with resistance to numerous drugs or small molecules in STAD.

**Conclusions:**

We identified CXCL2 as a novel therapeutic target and associated with immune infiltration in STAD.

## 1. Introduction

Gastric cancer (GC) is a malignant tumor originating from the epithelium of the gastric mucosa, ranking 5th most common in the incidence of malignant tumors and 3rd most common in the death rate of cancer worldwide.^[1,2]^ More than 95% of GC cases are stomach adenocarcinoma (STAD). With advances in the diagnosis and treatment of GC, the success rate of early GC treatment has improved,^[3]^ but the prognosis for patients with advanced GC remains poor, with a 5-year survival rate of <30%.^[1]^ Moreover, the overall survival rate for patients with advanced or metastatic gastric cancer is only about 12 months.^[4]^ With the development and application of high-throughput sequencing, gene microarray, and plasmapheresis technologies, and public databases,^[5]^ it is necessary to identify some important genes in STAD to facilitate a better understanding of its pathogenesis, prognostic, which is important for improving treatment outcomes.

C-X-C motif (CXC) chemokines, a category of soluble proteins, can induce chemotaxis in tumor cells and other types of cells.^[6]^ Ever-increasing evidences indicate that the interaction of CXC chemokines with their respective receptors affects various biological progresses in tumors, including proliferation, invasion, and metastasis, which can result in therapeutic failure.^[7,8]^ Moreover, CXC chemokines were demonstrated to be biomarkers for prognosis and drug targets in several types of cancers.^[9,10]^

CXCL2, a small secreted member of the CXC chemokine family, affects cell proliferation and apoptosis in hepatocellular carcinoma.^[6]^ In addition, Zhang et al has suggested that CXCL2 is a prognostic biomarker in bladder cancer.^[11]^ In STAD, CXCL2 and CXCL1 are found to be associated with cancer chemoresistance and metastasis.^[12]^ These results suggest that CXCL2 may also play a critical role in cancer. However, limited study had comprehensively and systematically explored the role of CXCL2 in STAD. Thus, we performed a comprehensive study on the expression of CXCL2 in STAD, and its possible role as a biomarker in diagnosis, prognosis, and as predictive marker for drug therapy. In our study, the expression, prognostic value, and clinical function of CXCL2 were analyzed using several online bioinformatics tools, including UALCAN, GEPIA, Kaplan–Meier plotter, GSCALite, TIMER, and LinkedOmics. The results of our study provide more information on the role of CXCL2 as a biomarker for prognosis and predicting therapeutic efficacy in STAD.

## 2. Materials and Methods

### 2.1. Expression analysis

The Cancer Genome Atlas Program (TCGA, https://portal.gdc.cancer.gov/) STAD dataset and GSE118916 and GSE79973 dataset were used to analyze CXCL2 expression in STAD. The TCGA STAD dataset was employed to detected CXCL2 expression and its expression was correlated with clinicopathological characteristics, such as age, gender, and tumor stage using student’s *t* test. The expression was normalized to transcripts per kilobase million (TPM) value before comparing expression of CXCL2 among different subgroups. *P* < .05 was considered statistically significant.

### 2.2. Prognostic analysis

Analysis of a possible prognostic role of CXCL2 in STAD was conducted with Kaplan–Meier plotter (https://kmplot.com) with TCGA STAD dataset, GSE62254 and GSE29272 dataset. In Kaplan–Meier plotter, subgroup prognosis analysis based on different clinicopathologic features and immune cells in STAD were perform using TCGA STAD dataset. *P* < .05 was considered statistically significant.

### 2.3. Specimens and patients of quantitative real time-polymerase chain reaction (qRT-PCR)

A total of 30 STAD tissues and normal gastric tissues were obtained from patients who underwent a tumor removal for STAD. Histological diagnosis and tumor grade were assessed by three experienced pathologists in accordance with 2010 American Joint Committee on Cancer staging system. All the patients don’t receive any local or systemic treatment preoperatively. Total RNA of STAD tissues and normal tissues were extracted with TRIzol reagent (Vazyme, Nanjing, China). The synthesis of cDNAs corresponding to the mRNAs of interest depended on PrimeScript RT-polymerase (Vazyme). qRT-PCR was performed using SYBR-Green Premix (Vazyme) with specific PCR primers (Sangon Biotech Co., Ltd, Shanghai, China). Glyceraldehyde-3-phosphate dehydrogenase was used as an internal control. The 2^−ΔΔCt^ method was used to calculate fold-changes. Primer sequences were as followed: GAPDH, Forward: GCACCGTCAAGGCTGAGAAC; Reverse: TGGTGAAGACGCCAGTGGA and CXCL2 forward: GCTTGTCTCAACCCCGCATC and CXCL2 reverse: TGGATTTGCCATTTTTCAGCATCTT. The difference of the expression of CXCL2 and the prognosis of CXCL2 in STAD were evaluated with Student’s *t* test and Kaplan–Meier analysis in GraphPad Prism7 software (GraphPad, Inc., La Jolla, CA).

### 2.4. Immune infiltrates analysis

The immune infiltrates analysis of CXCL2 in STAD was conducted with the Tumor Immune Estimation Resource (TIMER; https://cistrome.shinyapps.io/timer/), a tool for systematical analysis of immune infiltrates.^[13]^ In TIMER, CXCL2 was submitted to the “Gene” module for tumor-infiltrating immune cell (B cells, CD4+ T cells, CD8+ T cells, neutrophils, macrophages, and dendritic cells) analysis in invasive STAD. Moreover, CXCL2 was submitted to the “Correlation” module for immune cell biomarker analysis with “STAD” as the cancer type. These analysis were performed with Spearman correlation test using the TCGA STAD dataset. *P* < .05 was considered statistically significant.

### 2.5. Functional enrichment analysis

The functional enrichment analysis of CXCL2 in STAD was conducted with LinkedOmics, a tool for systematical analysis across TCGA cancers.^[14]^ We submitted CXCL2 to LinkedOmics and analyzed the significantly correlated genes with a false discovery rate (FDR) of 0.05 in TCGA STAD sample. Moreover, Gene Set Enrichment Analysis was used to explore the functions (gene ontology [GO] analysis and Kyoto Encyclopedia of Genes and Genomes [KEGG] pathways analysis) of CXCL2 in STAD with the minimum number of genes (Size) of 3 and the number of simulations set at 500. Several kinases, miRNAs and transcription factor (TF) targets of CXCL2 in invasive STAD were also identified with LinkedOmics. *P* < .05 was considered statistically significant.

### 2.6. Drug sensitivity analysis

The drug sensitivity analysis of CXCL2 in invasive STAD was conducted with GSCALite, a tool for systematical analysis across TCGA cancers.^[15]^ We submitted CXCL2 to GSCALite for drug analysis with the STAD TCGA datasets. A FDR < 0.05 was considered significant. In the drug sensitivity analysis, Spearman correlation was used to analyze the correlation between CXCL2 expression and 481 small molecules or drugs from the Cancer Therapeutics Response Portal (CTRP) and 265 small molecules or drugs from Genomics of Drug Sensitivity in Cancer (GDSC).

## 3. Results

### 3.1. CXCL2 expression in STAD

**Figure 1.**
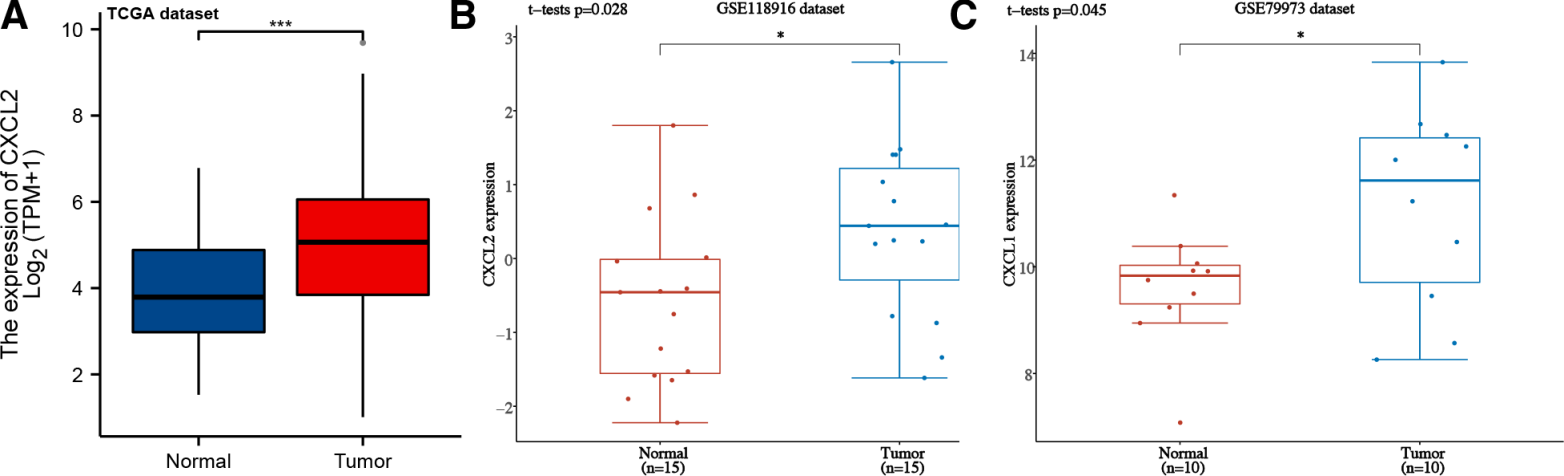
CXCL2 expression in STAD. Box plot showing CXCL2 levels in STAD tissues and normal STAD tissues in TCGA (A), GSE118916 (B), and GSE79973 (C) dataset. *P < .05, ***P < .001. CXC = C-X-C motif, STAD = stomach adenocarcinoma, TCGA = The Cancer Genome Atlas Program.

**Figure 2.**
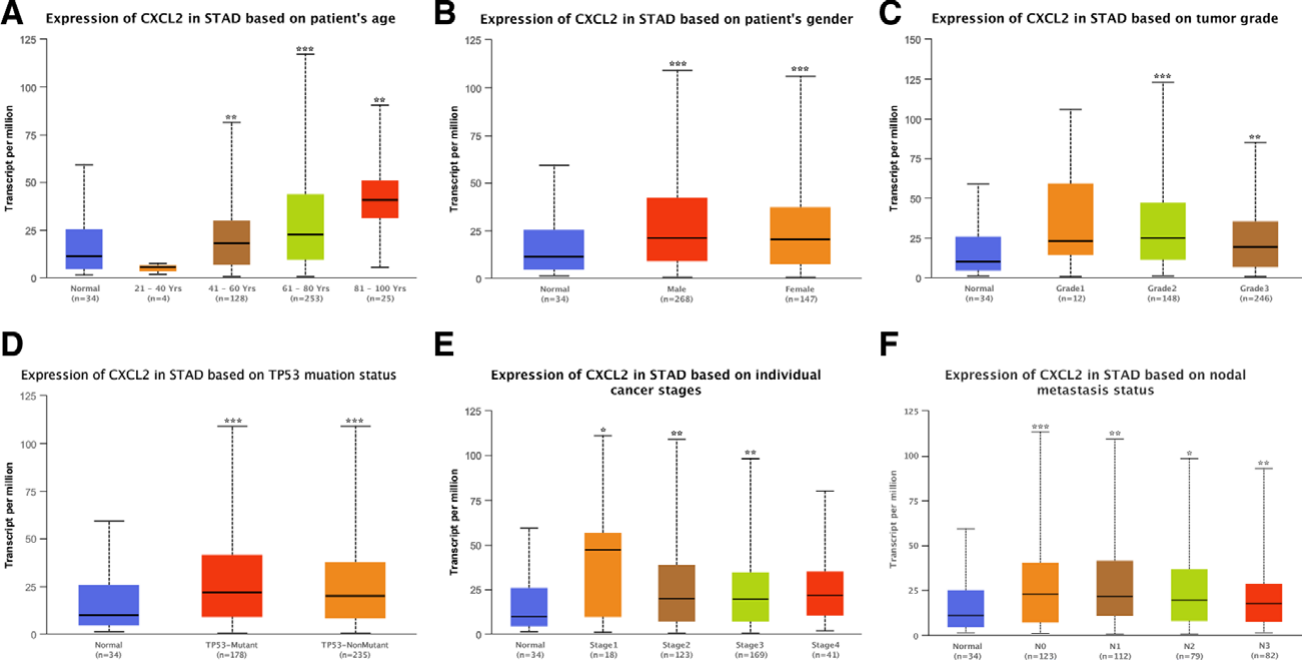
CXCL2 expression in subgroups of patients with STAD. CXCL2 expression in subgroups of patients with STAD stratified based on age (A), gender (B), tumor grade (C), TP53 mutation status (D), individual cancer stage (E), and nodal metastasis status (F). Data are mean ± SE. *P < .05, **P < .01, ***P < .001. CXC = C-X-C motif, STAD = stomach adenocarcinoma.

According to the results of TCGA, CXCL2 mRNA levels were significantly elevated in STAD (Fig. 1A, *P* < .001). Based on the data of GSE118916 (Fig. 1B, *P* = .028) and GSE79973 (Fig. 1C, *P* = .045) dataset, the expression of CXCL2 was also upregulated in STAD. Further sub-group analysis of multiple clinic pathological features was also conducted in TCGA STAD samples. Interestingly, CXCL2 levels were markedly upregulated in STAD patients compared with healthy volunteers in subgroup analysis based on age, gender, tumor grade, TP53 mutation status, individual cancer stage, and nodal metastasis status (Fig. 2A–F). Therefore, reduced CXCL2 expression may be a diagnostic biomarker for STAD.

### 3.2. The prognostic value of CXCL2 in STAD

**Figure 3.**
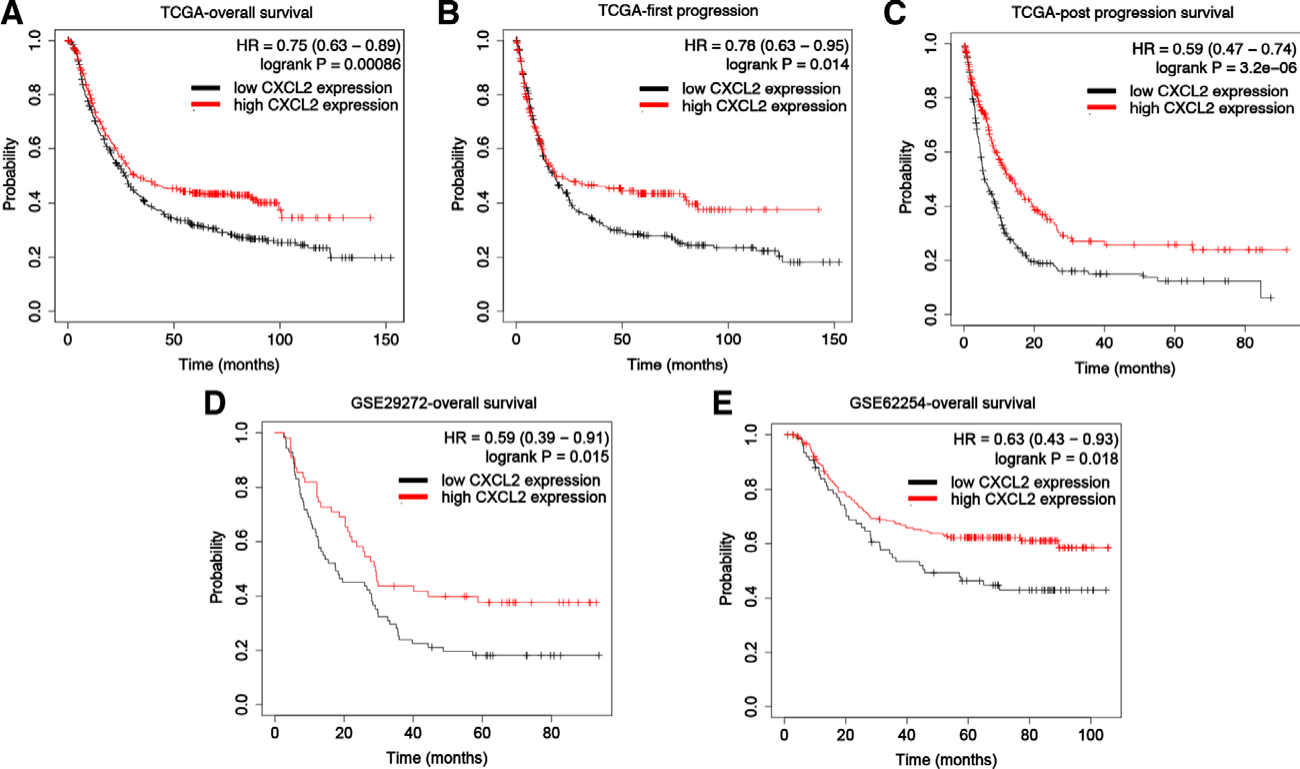
The prognostic value of CXCL2 in patients with STAD. The overall survival (A), first progression (B), and post progression survival (C) in patients with STAD patients with high and low expression of CXCL2 TCGA dataset. The overall survival in patients with STAD patients with high and low expression of CXCL2 in GSE29272 (D) and GSE62254 (E) dataset. CXC = C-X-C motif, HR = hazard ratio, STAD = stomach adenocarcinoma, TCGA = The Cancer Genome Atlas Program.

The data from TCGA indicated that the group of STAD patients with higher CXCL2 level had a better overall survival (*P* = .00086, hazard ratio [HR] = 0.75, Fig. 3A) compared with patients with low CXCL2 level. Moreover, high CXCL2 level was associated with better first progression (*P* = .014, HR = 0.78, Fig. 3B) and post progression survival (*P* = 3.2e-6, HR = 0.59, Fig. 3C) in STAD. In GSE29272 (*P* = .015, Fig. 3D) and GSE62254 (*P* = .018, Fig. 3E) datasets, STAD patients with high CXCL2 expression also had a prolonged overall survival. Therefore, CXCL2 is a promising novel prognostic marker for invasive STAD.

### 3.3. Building a predictive nomogram

**Figure 4.**
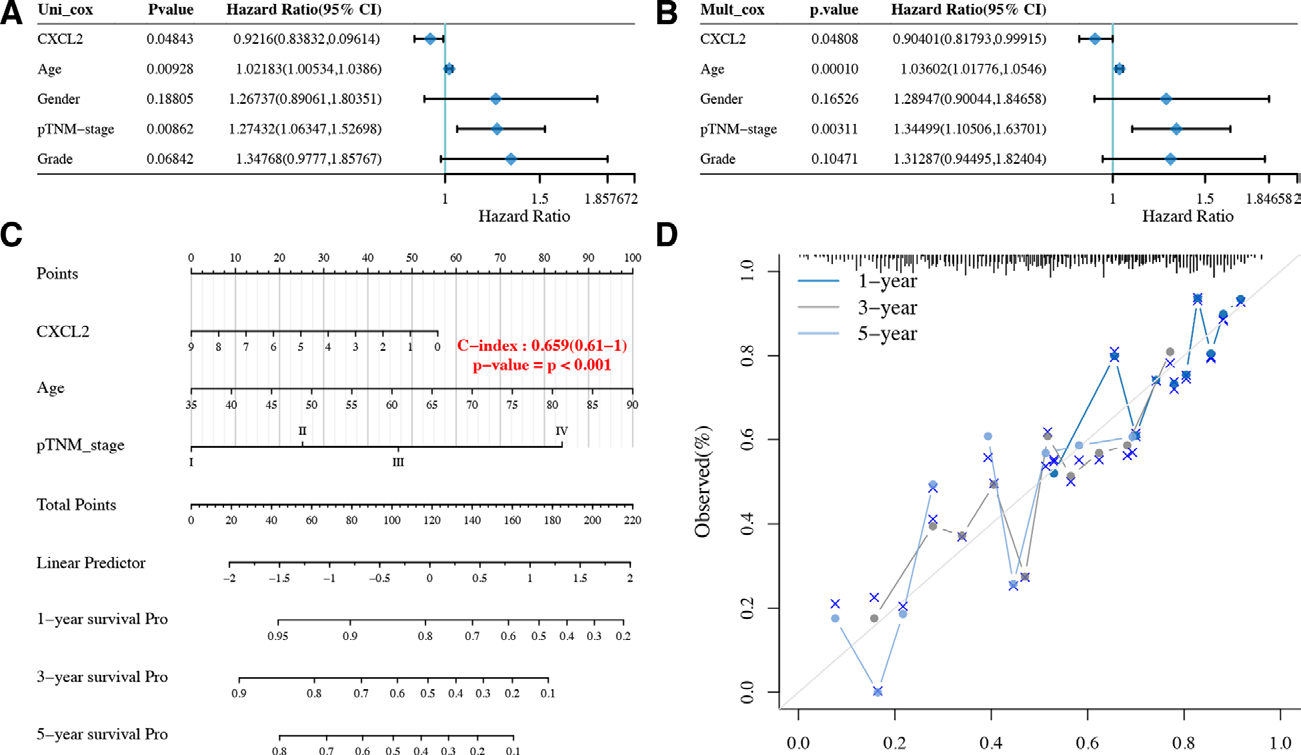
Univariate and multivariate cox regression of CXCL2 in STAD. (A and B) Hazard ratio and *P* value of constituents involved in univariate and multivariate cox regression and some patients’ parameters and CXCL2. (C and D) Nomogram to predict the 1-year, 3-year, and 5-year overall survival of STAD patients. Calibration curve for the overall survival nomogram model in the discovery group. A dashed diagonal line represents the ideal nomogram. CXC = C-X-C motif, STAD = stomach adenocarcinoma, TCGA = The Cancer Genome Atlas Program.

Considering clinicopathologic features and CXCL2 expression, We then resorted a nomogram to construct a predictive model that could predict the survival probability of STAD patient. The univariate and multivariate analysis revealed that CXCL2, pTNM stage and age were independent factors affecting the prognosis of STAD patients (Fig. 4A and B, all *P* < .05). We generated a nomogram to predict the 1-year, 3-year, and 5-year overall survival (OS) rates in the discovery group using the cox regression algorithm (Fig. 4C). The calibration plots for 1-year, 3-year, and 5-year OS rates were predicted relatively well compared with an ideal model in the entire cohort (Fig. 4D).

### 3.4. Validation of the expression and prognostic value of CXCL2 in STAD

**Figure 5.**
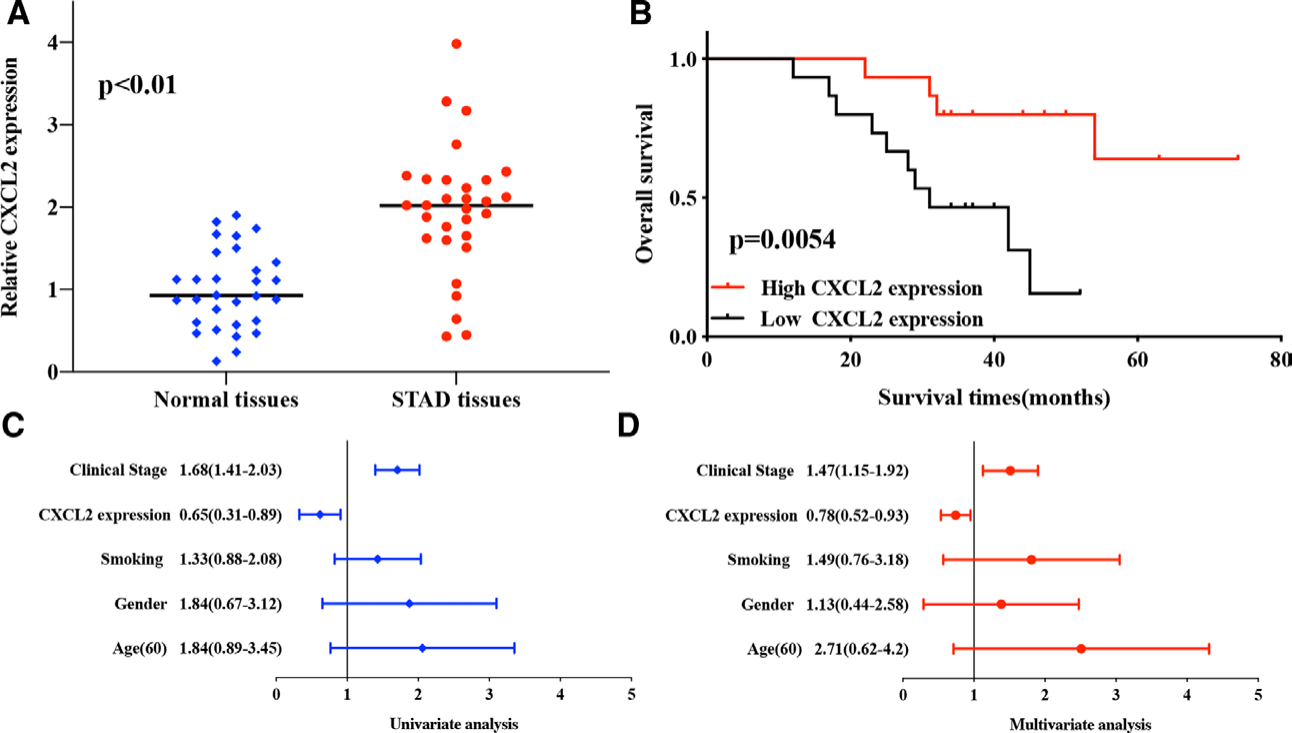
Validation of the expression and prognostic value of CXCL2 in STAD. (A) The relative expression of CXCL2 in STAD tissues and normal tissues. (B) The overall survival in patients with STAD patients with high and low expression of CXCL2. (C and D) Univariate and multivariate cox regression of CXCL2 and clinical characters in STAD. CXC = C-X-C motif, STAD = stomach adenocarcinoma.

We then verified the expression and prognostic value of CXCL2 in STAD. As expected, the resulted revealed that CXCL2 expression was lower in STAD tissues compared with normal tissues (Fig. 5A, *P* < .01). Moreover, prognosis analysis revealed that STAD patients with high CXCL2 level had a better overall survival (Fig. 5B, *P* = .0054). Univariate and multivariate analysis revealed that CXCL2 expression and clinical stage were factors affecting the prognosis of STAD patients (Fig. 5C and D). These results were consistent with above data.

### 3.5. Immune infiltrate analysis of CXCL2 in STAD

**Figure 6.**
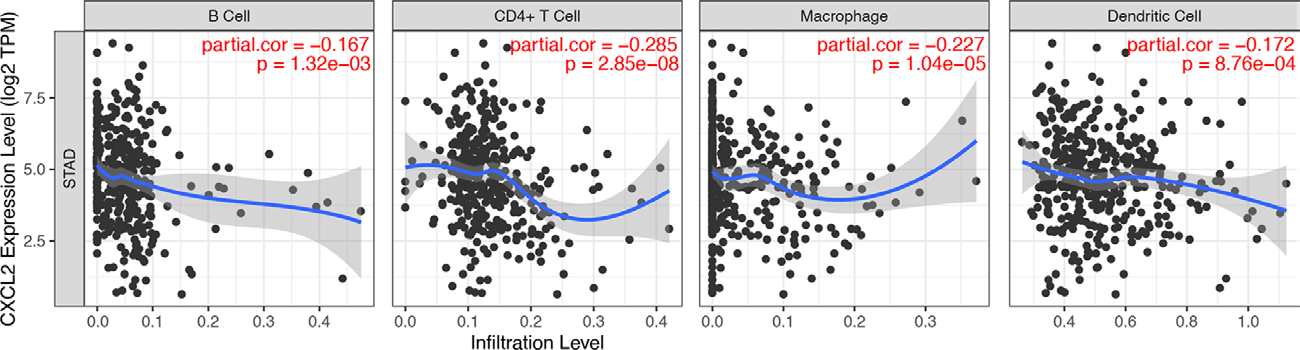
The correlation between CXCL2 level and immune cell infiltration in STAD. CXC = C-X-C motif, STAD = stomach adenocarcinoma.

Immune infiltrate analysis of CXCL2 in STAD was conducted with TIMER. The results are shown in Figure 6. We found that CXCL2 expression showed a negative correlation with immune infiltrates levels of B cells (Cor = −0.167, *P* = 1.32e-03), CD4+ T cells (Cor = −0.285, *P* = 2.85e-08), Macrophage (Cor = −0.227, *P* = 1.04e-05) and dendritic cells (Cor = −0.172, *P* = 8.76e-04). Moreover, immune-related biomarkers analysis demonstrated significant association between CXCL2 levels and the level of some gene biomarkers in STAD (Table 1). Specifically, expression level of the gene biomarkers of CD8+ T cells (D8A, CD8B), B cells (CD19, CD79A), Monocytes (CD115), and M1 Macrophage (NOS2, IRF5, and PTGS2) (CCL2, IL10) were significantly correlated with CXCL2 expression. The levels of KIR2DL4, IFNG, and TNF were positively associated with CXCL2 level in STAD. The result also suggested significant correlation between CXCL2 expression and the expression of GATA3, STAT3, IL17A, STAT5B, TGFB1, CTLA4, and GZMB. Therefore, our results indicated a significant role for CXCL2 in tumor immune escape. CXCL2 may act as a potential biomarker for immunotherapy and drug screening in STAD.

**Table 1:** Correlation analysis between CXCL2 and gene biomarkers of immune cells in STAD (TIMER).

Description	Gene markers	Cor	*P* value
CD8+ T cell	CD8A	−0.105	^*^
	CD8B	−0.123	^*^
T cell (general)	CD3D	0.018	.719
	CD3E	−0.001	.988
	CD2	0.001	.998
B cell	CD19	−0.109	^*^
	CD79A	−0.103	^*^
Monocyte	CD86	0.012	.8
	CD115(CSF1R)	−0.108	^*^
TAM	CCL2	0.029	.549
	CD68	−0.011	.831
	IL10	0.085	.085
M1 Macrophage	INOS (NOS2)	0.184	^***^
	IRF5	−0.1	^*^
	COX2(PTGS2)	0.376	^***^
M2 Macrophage	CD163	0.011	.818
	VSIG4	−0.047	.343
	MS4A4A	−0.114	^*^
Neutrophils	CD66b (CEACAM8)	0.031	.522
	CD11b (ITGAM)	−0.027	.59
	CCR7	−0.09	.066
Natural killer cell	KIR2DL1	0.043	.379
	KIR2DL3	0.047	.344
	KIR2DL4	0.164	^***^
	KIR3DL1	0.055	.262
	KIR3DL2	0.08	.103
	KIR3DL3	0.069	.16
	KIR2DS4	0.082	.096
Dendritic cell	HLA-DPB1	0.015	.763
	HLA-DQB1	0.06	.221
	HLA-DRA	0.076	.122
	HLA-DPA1	0.029	.55
	BDCA-1(CD1C)	−0.183	^***^
	BDCA-4(NRP1)	−0.041	.401
	CD11c (ITGAX)	0.067	.17
Th1	T-bet (TBX21)	0.003	.951
	STAT4	−0.031	.53
	STAT1	0.071	.149
	IFN-g (IFNG)	0.188	^***^
	TNF-a (TNF)	0.422	^***^
Th2	GATA3	−0.246	^***^
	STAT6	−0.048	.325
	STAT5A	−0.036	.47
	IL13	0.042	.391
Tfh	BCL6	−0.045	.359
	IL21	0.071	.15
Th17	STAT3	0.059	^*^
	IL17A	0.30	^***^
Treg	FOXP3	0.044	.369
	CCR8	0.023	.644
	STAT5B	−0.172	^***^
	TGFb (TGFB1)	−0.161	^**^
T cell exhaustion	PD-1 (PDCD1)	−0.04	.418
	CTLA4	0.126	^*^
	LAG3	0.05	.308
	TIM-3 (HAVCR2)	0.006	.911
	GZMB	0.202	^***^

CXC = C-X-C motif, STAD = stomach adenocarcinoma, TIMER = Tumor Immune Estimation Resource.

* *P* < .05.

** *P* < .01.

*** *P* < .001.

### 3.6. Prognostic analysis of CXCL2 expressions in STAD based on immune cells

**Figure 7.**
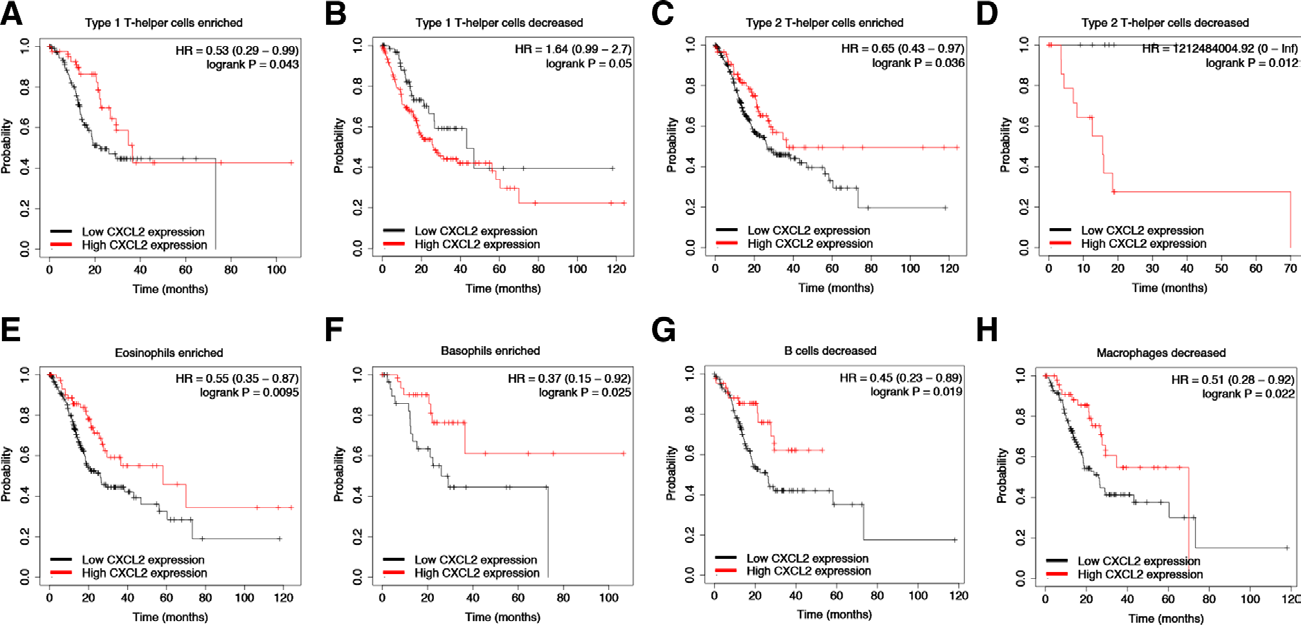
Prognostic value of CXCL2 in STAD based on different immune cells subgroups. The overall survival in patients with STAD patients with high and low expression of CXCL2 in enriched/decreased Type 1 T-helper cells cohort (A and B), enriched/decreased Type 2 T-helper cells cohort (C and D), enriched Eosinophils cohort (E), enriched Basophils cohort (F), decreased B cells cohort (G), decreased Macrophage cohort (H). CXC = C-X-C motif, HR = hazard ratio, STAD = stomach adenocarcinoma.

Above results found that CXCL2 expression was associated with the favorable prognosis and immune infiltration in CXCL2. In order to further verify whether CXCL2 expression affected the prognosis due to immune infiltration, we further conducted prognostic analysis of CXCL2 expressions in STAD based on immune cells using Kaplan–Meier plotter. As we could see in Figure 7, high CXCL2 expression of STAD in enriched Type 1 T-helper cells cohort (Fig. 7A), enriched/decreased Type 2 T-helper cells cohort (Fig. 7C and D), enriched Eosinophils cohort (Fig. 7E), enriched Basophils cohort (Fig. 7F), decreased B cells cohort (Fig. 7G), decreased Macrophage cohort (Fig. 7H) were associated with favorable prognosis. However, we also found that high CXCL2 expression of STAD in decreased Type 1 T-helper cells cohort (Fig. 7B) was associated with poor prognosis. Therefore, CXCL2 may affect the prognosis of STAD patients in part due to immune infiltration.

### 3.7. Enrichment analysis of CXCL2 in STAD

**Figure 8.**
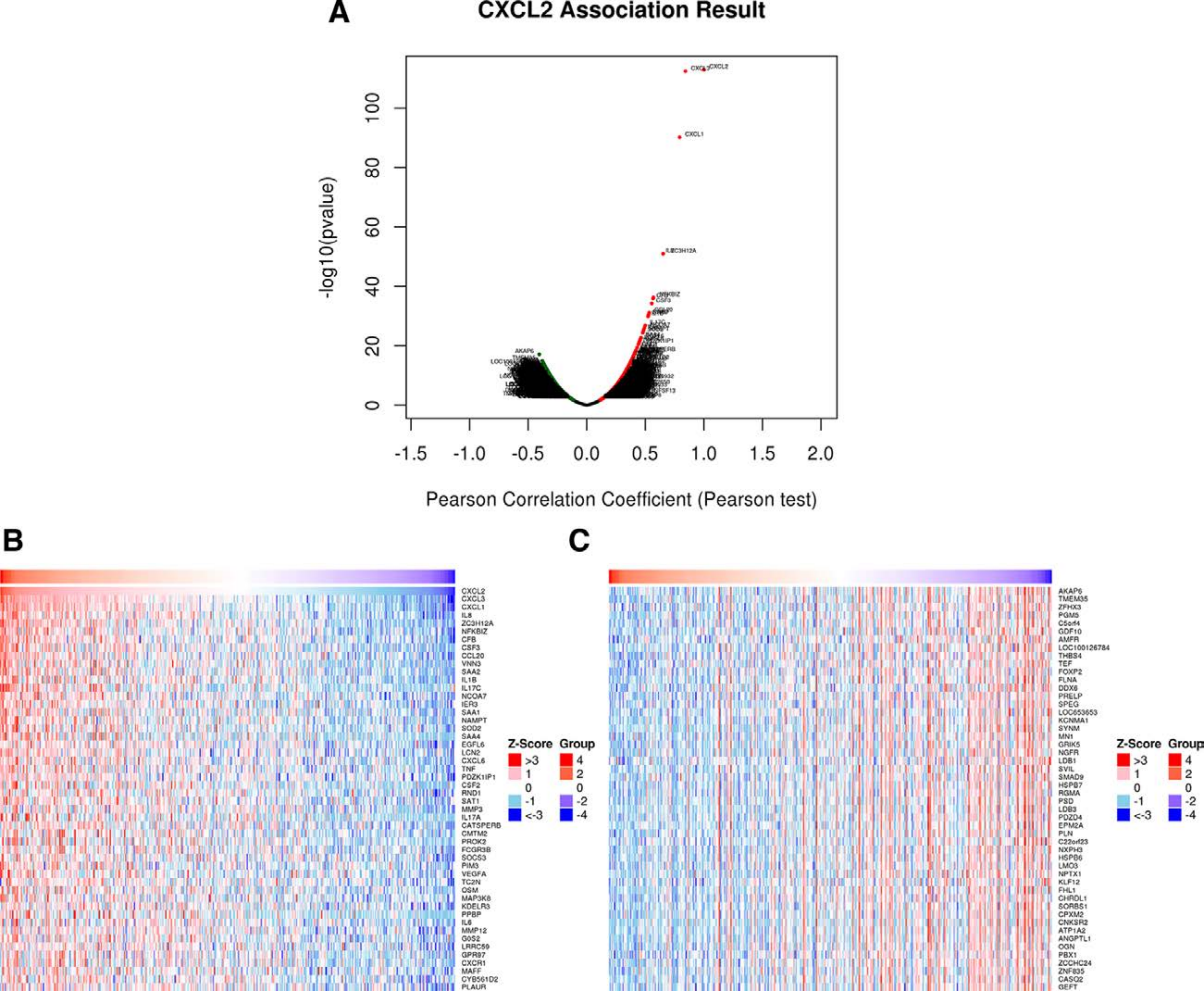
CXCL2-assocaited genes in STAD. (A) The genes significantly correlated with CXCL2 expression in STAD. (B and C) The top 50 genes positively or negatively correlated with CXCL2 in STAD in heat maps. CXC = C-X-C motif, STAD = stomach adenocarcinoma.

**Figure 9.**
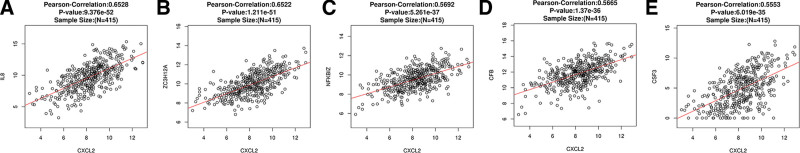
The top five significant genes correlated with CXCL2 in STAD. The correlation between CXCL2 and IL8 (A), ZC3H12A (B), NFKBIZ (C), CFB (D), and CSF3 (E) in STAD. CXC = C-X-C motif, STAD = stomach adenocarcinoma.

**Figure 10.**
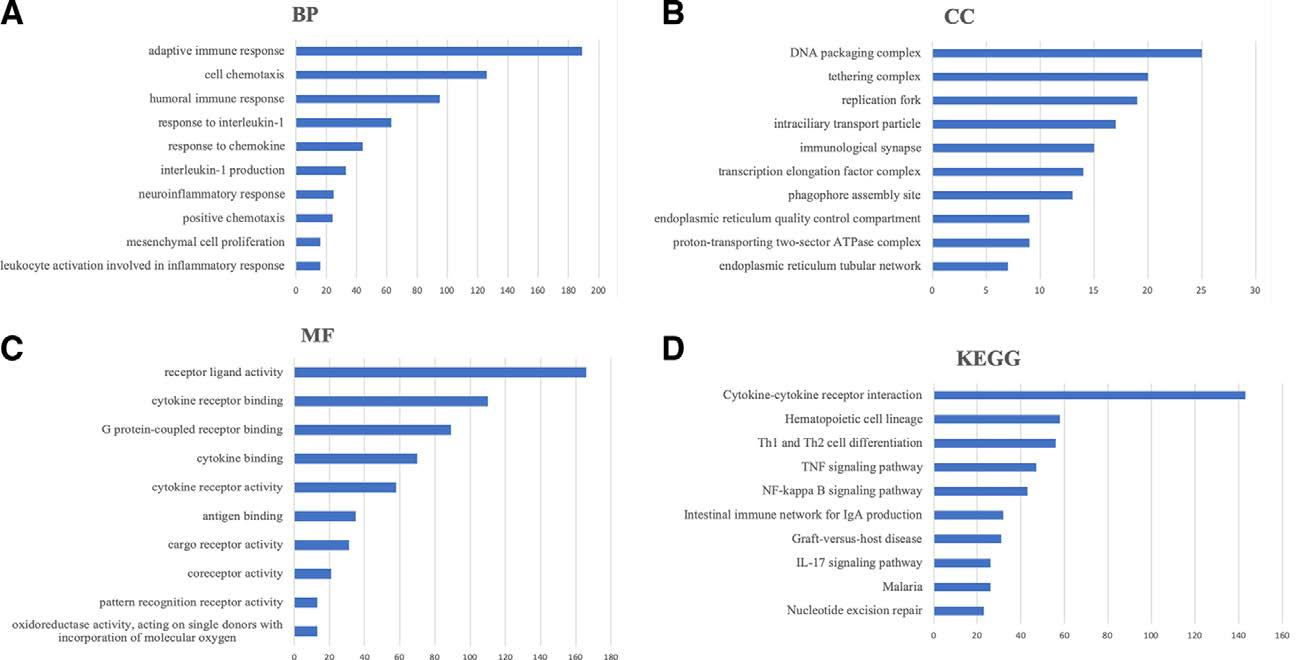
Enrichment analysis of CXCL2 in STAD. (A–C) GO analysis. (D) KEGG pathway analysis. BP = biological process, CC = cellular component, CXC = C-X-C motif, GO = gene ontology, KEGG = Kyoto Encyclopedia of Genes and Genomes, MF = molecular function, STAD = stomach adenocarcinoma.

In order to explore the function of CXCL2, we conducted a GO enrichment analysis of CXCL2 in STAD. We first explored CXCL2-associated genes and found that 14026 genes significantly associated with CXCL2 in STAD (Fig. 8A, *P* < .05). We also extracted the top 50 most significant genes that were positively or negatively associated with CXCL2 in STAD, as shown in Figure 8B and C. The top five most significantly CXCL2-associated genes were IL8, ZC3H12A, NFKBIZ, CFB, and CSF3 (Fig. 9A–E). The GO items shown in Figure 10A–C revealed that CXCL2 and its associated genes were enriched in leukocyte activation involved in immune response, humoral cell chemotaxis, inflammatory response, positive chemotaxis, interleukin-1 production, response to chemokine, DNA packaging complex, immunological synapse, receptor ligand activity, cytokine binding and receptor activity, and cytokine G protein-coupled receptor binding. Moreover, the KEGG analysis revealed that CXCL2 and its associated genes were enriched in Cytokine-cytokine receptor interaction, Th1 and Th2 cell differentiation, NF-kappa B signaling pathway, TNF signaling pathway, IL-17 signaling pathway, and nucleotide excision repair (Fig. 10D).

### 3.8. The CXCL2-associated kinase, miRNA and TF target in STAD

We also detected CXCL2-associated kinases, miRNAs and TF targets in STAD. Our results indicate that the top five most significant kinase targets of CXCL2 in STAD were MAPK11, MAPK12, LCK, MAPK13, and MAPK1 (Table 2). As for the miRNA targets of CXCL2 in STAD, the top five were enriched in MIR-345 (AGTCAGC), MIR-154 and MIR-487 (GTATGAT), MIR-452 (GAGACTG), MIR-216 (TGAGATT), and MIR-210 (ACGCACA) (Table 2). Moreover, the top five TF targets were V$SRF_01, V$STAT3_01, V$STAT5A_01, V$SRF_Q5_01, and V$NFKB_C (Table 2).

**Table 2:** The kinase and transcription factor-target networks of CXCL2 in STAD (LinkedOmics).

Enriched category	Geneset	Leading edge num	FDR
Kinase target	Kinase_ MAPK11	28	0
	Kinase_ MAPK12	23	0
	Kinase_ LCK	43	0
	Kinase_ MAPK13	20	0
	Kinase_ MAPK10	25	0
miRNA target	AGTCAGC, MIR-345	18	0
	GTATGAT, MIR-154, MIR-487	28	0
	GAGACTG, MIR-452	23	0
	TGAGATT, MIR-216	27	0
	ACGCACA, MIR-210	5	0.007
Transcription factor target	V$SRF_01	20	0
	V$STAT3_01	10	0
	V$STAT5A_01	87	0
	V$SRF_Q5_01	71	0
	V$NFKB_C	92	0

CXC = C-X-C motif, FDR = false discovery rate, STAD = stomach adenocarcinoma.

### 3.9. Drug sensitivity analysis of CXCL2 in STAD

**Figure 11.**
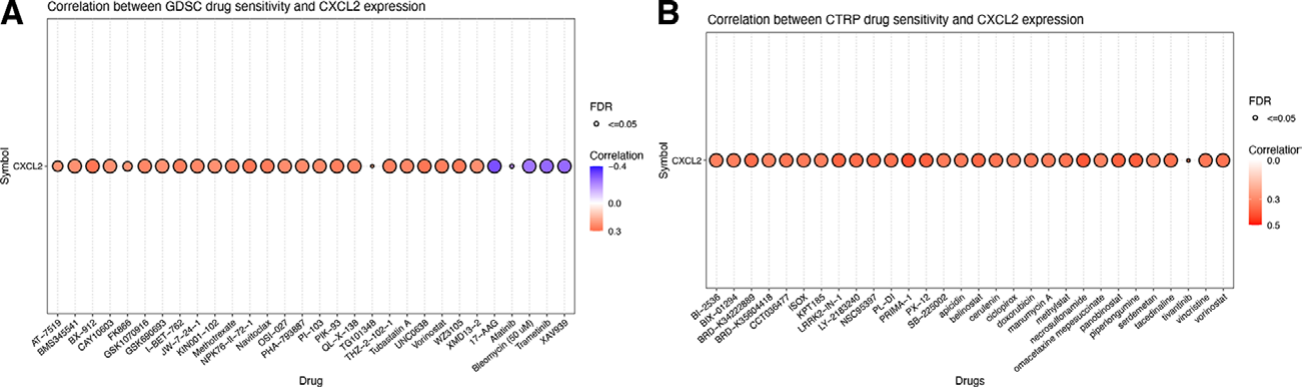
Drug resistance analysis of CXCL2 in STAD. The correlation between CXCL2 expression and small molecules or drugs from Genomics of Drug Sensitivity in Cancer (A) and Cancer Therapeutics Response Portal (B). CXC = C-X-C motif, CTRP = Cancer Therapeutics Response Portal, FDR = false discovery rate, GDSC = Genomics of Drug Sensitivity in Cancer, STAD = stomach adenocarcinoma.

CXCL2 was submitted to GSCALite for drug sensitivity analysis with GDSC/CTRP IC50 drug data. As shown Figure 11A and B, high expression levels of CXCL2 were associated with resistance to some drugs or small molecules in GDSC and CTRP. These results further demonstrated that CXCL2 was a promising therapy target in STAD.

## 4. Discussion

STAD still poses a major threat to the health and well-being of people all over the world. With advances in the diagnosis and treatment of GC, the success rate of early GC treatment has improved,^[3]^ but the prognosis for patients with advanced GC remains poor, with a 5-year survival rate of <30%.^[1]^ Moreover, the overall survival rate for patients with advanced or metastatic gastric cancer is only about 12 months.^[4]^ The identification of novel biomarkers for the diagnosis and prognosis, and for the prediction of response to therapy is critically needed in STAD. In our study, we explored the use of CXCL2 as a novel biomarker for prognosis and drug screening in STAD.

Previous studies have revealed that CXCL2 plays a significant role in several types of cancers. We speculated that CXCL2 could be a promising biomarker for STAD as well. We first analyzed the CXCL2 levels in STAD. As expected, the results of TCGA and GEO dataset indicated that CXCL2 was significantly upregulated in tumor tissues. Moreover, subgroup analysis, based on age, gender, tumor grade, TP53 mutation status, individual cancer stage, and nodal metastasis status suggested that CXCL2 was significantly upregulated in STAD tissues compared with normal tissues, and that CXCL2 is a promising a diagnostic biomarker for STAD. Interestingly, further analysis revealed that CXCL2 also may act as a prognostic biomarker for STAD. CXCL2 had been found to act as a biomarker in several types of cancers. In bladder cancer, CXCL2 was suggested to be a predictor of therapeutic effectiveness and as a potential therapeutic target.^[11]^ Renal cell carcinoma patients with downregulation of CXCL2 was associated with a significantly better prognosis.^[10]^ Therefore, we hypothesized that CXCL2 could act both as a diagnostic and as a prognostic biomarker in STAD.

Previous studies also reported the significant role of other CXC chemokines in the prognosis of STAD. Expression of CXCL9/10/11/17 mRNA may be a promising prognostic indicator for Epstein-Barr virus associated STAD patients.^[16]^ Another study revealed that CXCL9 was positively correlated with a better prognosis for STAD patients.^[17]^ Moreover, combinations of initial serum ENA78/CXCL5 and SDF-1/CXCL12 can serve as serum biomarker panels to predict the presence and distant metastasis of STAD.^[18]^ Qi et al suggested CXCL8 as a potential biomarker for predicting disease progression in STAD.^[19]^

The analysis of the immune infiltrates for CXCL2 in STAD indicated a negative correlation between CXCL2 levels in immune cells infiltrates and immune gene biomarker levels. Actually, immune infiltrates play a significant role in the tumor microenvironment.^[20,21]^ In addition, dysregulation of immune cells and immune gene biomarkers exerts an important role in mediating tumor immune escape, which could result in tumor progression and metastasis.^[22–24]^ Moreover, these immune cells and immune gene biomarkers could act as biomarkers for the prognosis or therapy of various types of cancers. A high level of activated T cells and an increased abundance of dendritic cells predicted a better prognosis in cutaneous melanoma.^[25]^ PD-1-expressing T Cells act as a biomarker predicting infusive prognosis in HPV-associated head and neck cancer.^[26]^ Therefore, CXCL2 may play a significant role in tumor immune escape, and CXCL2 may act as a potential biomarker for immunotherapy and drug screening in STAD.

We also conducted a GO enrichment analysis of CXCL2 in STAD. In our study, enrichment analysis and cancer-related pathway of CXCL2 in STAD suggested that CXCL2 is mainly involved in leukocyte activation, immune response, immunological synapse, cytokine-cytokine receptor interaction, Th1 and Th2 cell differentiation, NF-kappa B signaling pathway, TNF signaling pathway, IL-17 signaling pathway, nucleotide excision repair, apoptosis pathway, EMT pathway, RAS/MAPK pathway, cell cycle pathway, DNA damage response pathway, and hormone AR pathway. Previous studies had revealed that these functions and pathways were involved in the immune response and the pathogenesis and progress of cancers. The NF-kB family is crucial for immune responses and inflammation and NF-kB has been implicated in the initiation, progression, and resistance to treatment in human cancers.^[27]^ Dysregulation of IL-17 signaling is related to immunopathology and tumor progression.^[28]^ The highly conserved RAS/MAPK signaling pathway plays an important role in various biological processes, such as invasion, apoptosis, and metastasis.^[29]^ Thus, CXCL2 may affect the tumorigenesis and progress of STAD via these signaling pathways.

We also identified several kinases, miRNAs and transcription factor targets of CXCL2 in STAD. Interestingly, we found that the kinase targets were involved in the regulation of cell proliferation, cell cycle control, apoptosis, and DNA repair.^[30,31]^ Drug sensitivity analysis represented another important part of our study, revealing that high expression of CXCL2 was associated with resistance to numerous drugs or small molecules in GDSC and CTRP. CXCL1 and CXCL2 are 90% identical by amino acid sequence and signal through the same receptor, CXCR2.^[32]^ Therefore, further studies should be performed to verify whether CXCL2 could act as a biomarker for drug screening in STAD.

There is no doubt that our study also had some limitations. First, our study only focused on the role of CXCL2 in STAD. Several other chemokines may be also involved in the development, progression and progression of STAD. Moreover, further study should be performed to clarify the molecular mechanism of CXCL2 in STAD.

## 5. Conclusion

We identified CXCL2 as a novel potential prognostic biomarker and associated with immune infiltration in STAD, providing additional evidence for the clinical application of CXCL2.

## Acknowledgments

We thank Zhiwu Han for helping us with the qRT-PCR experiment. This study was approved by the Ethics Committee of Affiliated People’s Hospital of Jiangsu University. And all patients provided informed consent.

## Author contributions

**Data curation:** Junbo Zuo, Zhenhua Huang.

**Formal analysis:** Xuefeng Bu.

**Investigation:** Jingxin Zhang, Xuefeng Bu.

**Methodology:** Junbo Zuo.

**Project administration:** Xuefeng Bu.

**Resources:** Zhenhua Huang.

**Supervision:** Zhenhua Huang.

**Validation:** Jingxin Zhang, Xuefeng Bu.

**Visualization:** Wenji Hou.

**Writing – original draft:** Jingxin Zhang, Xuefeng Bu.

**Writing – review & editing:** Wenji Hou, Xin Ding, Xuefeng Bu.
